# 522. Significant Improvement in Health-Related Quality of Life (HRQL) with RBX2660: Results from a Phase 3 Randomized, Placebo-Controlled Trial in Recurrent *Clostridioides Difficile* Infection (PUNCH CD3)

**DOI:** 10.1093/ofid/ofac492.577

**Published:** 2022-12-15

**Authors:** Paul Feuerstadt, Erik R Dubberke, Amy Guo, Adam Harvey, Min Yang, Viviana García-Horton, Mirko Fillbrunn, Glenn S Tillotson, Lindy Bancke, Kevin W Garey

**Affiliations:** Yale University School of Medicine/PACT-Gastroenterology Center, Westport, Connecticut; Washington University, Saint Louis, Missouri; Ferring Pharmaceuticals, parsippany, New Jersey; Ferring Pharmaceuticals, parsippany, New Jersey; Analysis Group, Inc., Boston, Massachusetts; Analysis Group, New York City, New York; Analysis Group, Inc., Boston, Massachusetts; GST Micro LLC, NORTH, Virginia; Rebiotix, a Ferring Company, Roseville, Minnesota; University of Houston, Houston, TX

## Abstract

**Background:**

Recurrence of *Clostridioides difficile* infection (rCDI) is common - up to 35% of patients may recur. RBX2660 is a microbiota restoration therapy to reduce rCDI. Here we report 8 weeks HRQL results using the *Clostridioides difficile* Health-related Quality-of-Life Questionnaire (Cdiff32), a disease-specific instrument, from PUNCH CD3 (a randomized, double-blinded, placebo-controlled RBX2660 Phase 3 trial, NCT03244644).

**Methods:**

Cdiff32 includes three domains (physical, mental, and social) and a total score (all range from 0 to 100 [100 best possible]). Changes in Cdiff32 from baseline to week 8 were compared between RBX2660 and placebo (PBO) using unadjusted and adjusted analyses controlling for baseline score, demographic and disease characteristics. Per trial protocol, missing data were imputed via last observation carried forward (LOCF); as-observed data were also analyzed. Patients experiencing recurrence after blinded treatment received open-label RBX2660 per physician discretion; these participants were excluded unless, per LOCF, data were available from the blinded period for week 8 use.

**Results:**

A total of 206 patients (140 RBX2660, 66 PBO) were included, with similar age (mean±SD) 61.1±16.9 yrs (RBX2660) and 57.3±16.4 yrs (PBO) and baseline Cdiff32 scores. More than half of the patients had multiple comorbidities. Cdiff32 scores improved significantly from baseline to weeks 1, 4, and 8 for both arms, with greater improvements for RBX2660 through week 8 (Figs. 1 & 2). At week 8, statistical differences were found for mental domain (unadjusted: 8.01±3.64; adjusted: 7.07, 95% confidence interval: [0.28, 13.86], both P< 0.05) and total score (adjusted: 6.11, [0.14, 12.08], P< 0.05), all favoring RBX2660. Results were similar for the as-observed analyses, with the adjusted physical domain also statistically favoring RBX2660.

**Figure:**
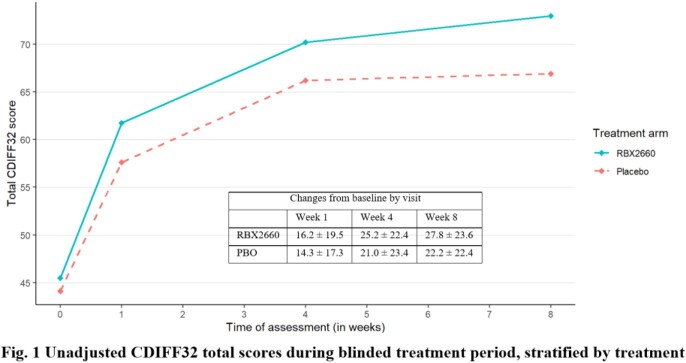

**Figure:**
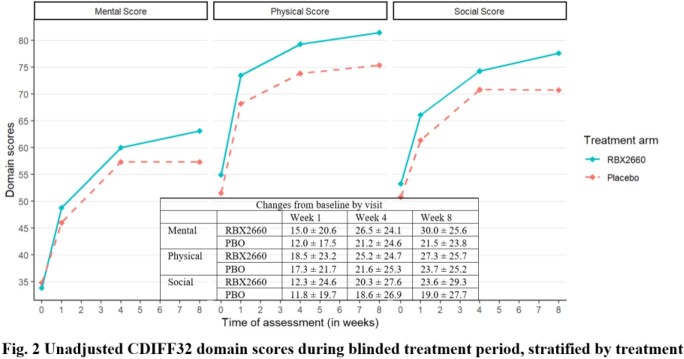

**Conclusion:**

Most patients in this study reported improved HRQL. Improvements were observed in both arms, but RBX2660-treated patients had more robust and sustained improvements with statistically significant differences in Cdiff32 scores. This study suggests that microbiome restoration therapy might positively affect HRQL; future research may link these improvements directly with microbiota changes.

**Disclosures:**

**Paul Feuerstadt, MD, FACG, AGAF**, Ferring/Rebiotix Pharmaceuticals: Advisor/Consultant|Ferring/Rebiotix Pharmaceuticals: Grant/Research Support|Merck and Co: Advisor/Consultant|SERES Therapeutics: Advisor/Consultant|SERES Therapeutics: Grant/Research Support|Takeda Pharmaceuticals: Advisor/Consultant **Erik R. Dubberke, MD, MSPH**, Abbott: Advisor/Consultant|Ferring: Advisor/Consultant|Ferring: Grant/Research Support|Merck: Advisor/Consultant|Pfizer: Advisor/Consultant|Pfizer: Grant/Research Support|Seres: Advisor/Consultant|Summit: Advisor/Consultant|Synthetic Biologics: Grant/Research Support **Amy Guo, PhD**, Ferring Pharmaceuticals: Employee **Adam Harvey, PhD**, Ferring Pharmaceuticals: Employment **Min Yang, MD, PhD**, Analysis Group, Inc.: I am an employee of Analysis Group, Inc., which has received consulting fees from Ferring for the conduct of this study. **Viviana García-Horton, PhD**, Analysis Group, Inc.: Employee of Analysis Group, Inc., which received consulting fees from Ferring for the conduct of this study. **Mirko Fillbrunn, PhD**, Analysis Group, Inc.: I am an employee of Analysis Group, Inc., which has received consulting fees from Ferring for the conduct of this study. **Glenn S. Tillotson, PhD**, Ferring Pharmaceuticals: Advisor/Consultant|Paratek Pharmaceuticals: Grant/Research Support|Spero Pharmaceuticals: Advisor/Consultant|Taro Pharmaceuticals: Advisor/Consultant **Lindy Bancke, PharmD**, Rebiotix, a Ferring Company: Employee **Kevin W. Garey, PharmD, MS**, Acurx: Grant/Research Support|cidara: Advisor/Consultant|cidara: Grant/Research Support|Paratek: Grant/Research Support|Seres Health: Grant/Research Support|Summit: Grant/Research Support.

